# Developing and Strengthening the Current and Future MCH Public Health Workforce: Building Capacity, Aligning Systems and Addressing Emerging Challenges

**DOI:** 10.1007/s10995-022-03459-6

**Published:** 2022-07-14

**Authors:** Dorothy Cilenti, Michelle Menser Tissue, Leslie deRosset

**Affiliations:** 1 UNC Gillings School of Global Public Health, Chapel Hill, NC USA; 2 Maternal and Child Health Bureau, Rockville, MD USA

Current and emerging maternal and child health public health (MCH) professionals have faced unprecedented challenges over the past 2 years. The COVID-19 pandemic highlighted critical gaps in public health infrastructure and intensified inequities across MCH populations. A nationwide racial reckoning catapulted discussion of structural and systemic inequities into the forefront of public health dialogue and action. These challenges layered upon existing workforce shortages and infrastructure challenges. Taken together, they underscore the urgent need for increased investment in the current and future public health workforce and a need to better understand programs, policies and innovations that will support and advance the public health workforce.

In this special issue, we highlight the successes and needs of the MCH public health workforce, with a focus on current and future professionals that work in state maternal and child health agencies (Title V). Data from the 2017 Public Health Workforce Interest and Needs Survey (PH WINS) show that only 14% of the state and local governmental MCH workforce has received formal training in public health, 25% of the workforce plan to leave their position in the next year, and 22% intend to retire in the next 5 years (de Beaumont Foundation, 2017). Inadequate pay, lack of opportunities for advancement, and workplace environment were the top reason(s) for leaving their current position (de Beaumont Foundation, 2017). A qualitative analysis in 2018 among Title V Block Grant Application/Annual Reports also highlights challenges with retention of qualified staff, an aging workforce, and ongoing barriers to accessing training (Health Resources and Services Administration, 2022).

Despite these challenges, PH WINS data also highlight the dedication of the MCH workforce: more than nine in ten (96%) felt their work is important and 95% were determined to give their best every day (de Beaumont Foundation, 2017). Data also show a strong commitment to addressing social determinants of health (de Beaumont Foundation, 2017). The forthcoming release of 2021 PH WINS data will offer additional insights about the strengths and urgent needs of the MCH public health workforce, though preliminary findings reveal troubling trends in mental health, burnout and workforce retention. Despite these findings, initial data also show a commitment to the work and populations served remains (PH WINS, 2022).

While the multiple, intersecting challenges of the past 2 years have highlighted the significant challenges that lay ahead for public health systems and the workforce, it also presents an opportunity to highlight how a diverse and well-prepared MCH public health workforce can drive improvements in population health. This special issue of the Maternal and Child Health Journal helps to fill a gap in the peer-reviewed literature on the MCH public health workforce by sharing original research, commentaries, innovations, and policy and program evaluations that can advance efforts to develop the current and future MCH public health workforce.

Among the articles in this special issue are findings from Health Resources and Services Administration’s Maternal and Child Health Bureau-funded undergraduate and graduate training programs across the United States. These programs highlight successes and opportunities to create equitable and diverse pathways for the future public health workforce. Examples of best practices to train and mentor undergraduate and graduate students, as well as the development of public health curricula grounded in equity and anti-racism, are shared.

As public health continues to grapple with the COVID-19 pandemic, several articles stress the need and opportunities for building leaders in MCH who can address emerging challenges. Strategic planning, leadership development, and innovative programmatic opportunities are highlighted.

The National MCH Workforce Development Center and the Maternal Health and Learning Innovation Center provide qualitative and quantitative data from evaluations with Title V agency staff, individuals with lived experience, and public health partners regarding the impact of these Centers in enhancing Title V MCH workforce capacity to address complex challenges.

The Maternal and Child Health Bureau released a new strategic plan in 2021 to guide work over the next 10–15 years. Goal 3 (*Strengthen Public Health Capacity and Workforce for MCH*) supports the future of the MCH public health workforce with 13 recommendations. These recommendations are described in the Ramos et al. article (2022) included in this special issue, and discuss the trajectories for MCH public health careers, high-quality training, and how to build the MCH workforce capacity and skills to address inequities and social determinants of health. Additional recommendations address the need to build a diverse workforce, increase community engagement, and mobilize people with lived experiences in service to MCH populations.

So, what is the desired future state of the MCH public health workforce in 10–15 years? The manuscripts in this special issue seem to suggest that the MCH public health workforce will need to have the following characteristics:

- Diversity that mirrors the populations and communities served
- Competency in policy development and implementation
- Ability to work collaboratively across systems and sectors
- Meaningful partnerships with those with lived experiences to address the social determinants of health
- Razor-sharp focus on equity
- Mentors for future MCH professionals
- Competency in translating research and evidence into practice

To progress to this future state, we will need to acquire better workforce data to assess the extent to which we are moving towards these goals. The field of MCH specifically, and public health in general, needs to benchmark the composition of the workforce, the skills and competencies of individuals working in the field, and the ability of the workforce to carry out the core functions and essential services of public health (PHNCI, 2020). Moreover, beyond foundational skills, we need to build a state of the art, twenty-first century workforce that leverages the experiences of people living in communities disproportionately impacted by illness and death and tailors and adapts public health approaches for the precise benefit of those communities.
